# Effect of Aging and Cortical Stroke on Motor Adaptation to Overground Gait-Slips: Quantifying Differences in Adaptation Rate and Adaptation Plateau

**DOI:** 10.3390/biomechanics3010003

**Published:** 2023-01-05

**Authors:** Rudri Purohit, Shuaijie Wang, Tanvi Bhatt

**Affiliations:** 1 Department of Physical Therapy, College of Applied Health Sciences, University of Illinois, Chicago, IL 60612, USA; 2 PhD program in Rehabilitation Sciences, College of Applied Health Sciences, University of Illinois, Chicago, IL 60612, USA

**Keywords:** reactive balance, stability, motor adaptation, fall prevention, stroke, aging

## Abstract

We examined the effect of aging and cortical stroke on the rate of motor adaptation (adaptation rate) and amount of performance gains (adaptation plateau) in balance skills. Fourteen older (≥60 years) and fifteen younger (<60 years) adults with chronic stroke, and thirteen healthy older adults (≥60 years) participated. Participants experienced 8 consecutive gait-slips (≤45 cm) to their non-paretic/dominant limb. Slip outcome (backward/no balance loss) was compared using generalized estimating equations (GEE). Proactive (pre-slip stability) and reactive adjustments (post-slip stability, slip displacement and velocity, and compensatory step length) were compared using non-linear regression models. GEE showed the main effect of group, trial, and group × trial interaction for slip outcome (*p* < 0.05). There were no differences in the adaptation rate for proactive and reactive variables and plateau for proactive variables (*p* > 0.05). However, both stroke groups demonstrated a smaller adaptation plateau for the majority of reactive variables compared to healthy older adults (*p* < 0.05). The rate of adaptation to gait-slips does not slow with aging and cortical stroke; however, cortical stroke, age notwithstanding, may reduce performance gains in reactive balance skills, possibly hindering retention and transfer to real-life scenarios. People with stroke may need adjunctive therapies/supplemental agents to apply laboratory-acquired balance skills to daily life.

## 1. Introduction

About 800,000 Americans suffer from stroke annually [1–3], resulting in varying degrees of sensorimotor dysfunction, gait, and balance impairments. These impairments are postulated to be associated with twofold higher fall-risk in individuals with stroke compared to healthy counterparts [4–6]. Moreover, fall incidence is higher during chronic phases of stroke recovery when individuals achieve community ambulation and are frequently exposed to environmental perturbations such as slips or trips [6,7]. Despite the well-established consequences of falls, current conventional interventions, including but not limited to dynamic balance and muscle strength training have reported limited reductions in fall-risk or incidence in people with chronic stroke [8–11]. Nevertheless, emerging alternative interventions, such as task-specific paradigms, have tested and established the preserved ability to relearn motor and balance skills during subacute and chronic phases of stroke recovery [12,13].

Perturbation-based balance training (PBT), consisting of repeated exposures to unpredictable perturbations (slips or trips) [14–17], is a task-specific intervention that improves reactive balance control. Reactive balance control is the central nervous system (CNS)’s ability to execute compensatory responses to maintain or regain stability following unpredicted perturbations potentially altering the relationship between the center of mass (COM) and the base of support (BOS) [18–20]. Especially, compensatory stepping responses are crucial to avoid falls from large-magnitude unpredictable environmental perturbations [21–23]. Systematic reviews in healthy older adults reported that even a single session of PBT can result in enhanced reactive balance control resulting in subsequent reductions in balance losses and a lower fall-risk [24–26]. A single session of PBT has shown to induce rapid motor adaptation during gait perturbations [16,27], and a long-term retention of the acquired skills (up to 12-months post-training) in healthy older adults [16,28,29]. Owing to the promising effects of PBT for fall prevention in healthy older adults, studies have examined its effect on motor skill acquisition and fall-risk reduction in people with chronic stroke [30–33].

In individuals with chronic stroke, perturbation-based interventions have reported immediate gains in motor performance and improvements in compensatory stepping responses with a single session of treadmill-delivered stance PBT [15,30,31,34]. Given the higher fall-risk during functional activities such as walking, incorporating paradigms that deliver perturbations mimicking real-life situations would be more task-specific. More recently, Dusane and Bhatt [33] reported reductions in balance losses and improvements in COM stability during pre- and post-slipping instances following eight consecutive overground gait-slips delivered to the non-paretic limb. However, this study did not quantify the rate or characteristics of motor adaptation during the training session. Previous studies in healthy young and older adults have indicated that rapid and maximal improvements in motor performance occur during the beginning of a training block (within five slips), which is followed by a steady state (plateau) achieved by the end of the block [27,35]. However, it is still unknown whether people with chronic stroke can demonstrate similar adaptation characteristics during gait-slips. Motor adaptation could be quantified by the rate at which the steady state can be attained (adaptation rate) and the level of achieving the steady state (adaptation plateau). Analysis of such characteristics might help understand the effect of cortical stroke on adaptation ability and the acquisition of fall-resisting skills through PBT.

About 75% of Americans with incident stroke are older adults (>60 years of age) [36,37], thus, aging is a common non-modifiable risk factor for stroke occurrence [38,39]. Our recent study compared reactive balance performance between older adults with chronic stroke and their younger counterparts during a novel, unpredicted gait-slip [40]. Compared to younger adults with chronic stroke, older adults exhibited impaired slipping limb control (i.e., higher slip displacement and faster slip velocity) which resulted in reduced post-slip COM stability and more falls during a novel gait-slip to the non-paretic limb [40]. While older adults with chronic stroke demonstrated higher fall-risk compared to younger counterparts, it is still unclear whether the greater deficits in reactive balance control in older adults with chronic stroke would limit their ability to acquire performance gains in fall-resisting skills. Clinically, examining the influence of aging and stroke on motor adaptation could help establish the optimal training dosages and propose effective fall-prevention strategies, particularly in older adults with chronic stroke.

Therefore, the purpose of this study was to examine whether aging with cortical stroke could affect motor adaptation during exposures to a block of overground slips delivered to non-paretic limb during walking. Firstly, we hypothesized that older adults with chronic stroke would display a slower rate of reduction in balance losses resulting from slower improvements (*adaptation rate*) in proactive and reactive variables compared to healthy older adults and young adults with chronic stroke receiving the same number of training trials. Proactive variables including pre-slipping COM stability and reactive variables including post-slipping COM stability, non-paretic slipping limb control (i.e., slip displacement and velocity), and paretic stepping abilities (i.e., compensatory step length). Second, we hypothesized that older adults with chronic stroke would demonstrate lower improvements in proactive and reactive balance variables after they reach a steady state of motor adaptation (*adaptation plateau*) compared to their younger and healthy counterparts.

## 2. Materials and Methods

### 2.1. Participants

In total, 15 community-dwelling younger adults with chronic stroke (<60 years), 15 older adults with chronic stroke (≥60 years), and 15 healthy older adults (≥60 years) were included in this study. For both stroke groups, people with stroke onset > 6 months confirmed by their physician were included. All included participants were able to ambulate independently for at least 10 meters with or without an assistive device. Participants were excluded if they exhibited (1) cognitive impairments (Montreal Cognitive Assessment score of ≤26/30); (2) speech impairments (Mississippi aphasia screening test score of ≤71/100); (3) poor bone density (T score of <−2 on heel ultrasound); (4) any other untreated/uncontrolled musculoskeletal, neurological or cardiopulmonary conditions; (5) loss of lower limb protective sensations (inability to perceive the 5.07/10 g on Semmes–Weinstein Monofilament [41–43]); (6) visual impairments (visual acuity assessment using Snellen’s chart with regular corrective glasses); or (7) inability to follow instructions due to cognitive or hearing deficits. Three participants were further excluded from data analysis. One older adult with chronic stroke and one healthy older adult was excluded from data analysis as there was improper (half) landing of slipping foot during the initial slips. Another healthy older adult was excluded from data analysis as the participant dropped out of the study after the initial two slips. Baseline clinical gait and balance measures including the Berg Balance Scale, Timed Up-and-Go Test, and 10-Meter Walk Test were assessed. We statistically matched young and older adults with chronic stroke based on motor impairment using the Chedoke–McMaster Stroke Assessment scale (CMSA Leg). Further, we statistically matched older adults with chronic stroke and healthy older adults based on their age. The demographic characteristics of all included participants are presented in Table 1. Prior to subject enrollment, the study was approved by the institutional review board of University of Illinois at Chicago. All participants included in the study provided written informed consent for the research experiment.

### 2.2. Experimental Setup

Experimental setup consisted of a customized 7 meter walkway with a pair of low-friction, computer-controlled sliding platforms mounted to supporting frames via linear ball bearings [16,44,45]. The supporting frame was bolted to a force plate (OR6-5-1000, AMTI, and Newton, MA, USA) to measure ground reaction forces (GRF) [16,27,33,35]. During unperturbed walking trials, the sliding devices were locked and embedded side-by-side and camouflaged with surrounding platform surfaces. During the perturbed walking trials, computer-controlled slips were delivered by unlocking of the sliding device immediately after detection of the slipping foot touchdown by the force plate [16,27,33]. Participants were safely secured in a full-body harness during the slip experiment.

### 2.3. Repeated-Slip Protocol

All participants first walked at their preferred walking speed with and without their assisted device for three trials each to get acquainted to the laboratory environment. Following six baseline walking trials, participants were alerted that a slip may occur, but without warning of the exact time and nature of the slip. The starting position for each participant was adjusted to ensure their slipping foot consistently landed on the desired sliding platform. Once the foot landing was attained, a sudden, unexpected slip (S1) was delivered to the non-paretic limb for young and older adults with chronic stroke and to the dominant limb for healthy older adults. This was followed by seven consecutive slips of similar nature (S2–S8).

### 2.4. Data Collection and Analysis

A 3-dimensional, 8-camera motion capture system (Qualisys Motion Capture System, Santa Rosa, CA, USA) was used to record full-body motion kinematics using a set of 30 retro-reflective markers (26 on bilateral bony landmarks, 2 on walkway, and 2 on movable sliders) [33,44,45]. Kinematic data sampled at 120 Hz was synchronized with the force plate and the load cell data that was collected at 600 Hz [33,44,45].

### 2.5. Outcome Measures

#### 2.5.1. Primary Outcome Measures

Primary outcomes including slip outcome, proactive and reactive balance adjustments were analyzed to examine the effect of age and cortical stroke on motor adaptation. Specifically, slip outcome was categorized as backward loss of balance or no loss of balance; proactive adjustments included pre-slip COM stability (COM position and velocity) at slipping limb touchdown. The reactive adjustments included post-slip COM stability (COM position and velocity) at the instance of non-slipping recovery limb touchdown, slip intensity (maximum slip displacement and maximum slip velocity) and compensatory step length. Secondary outcome measures included all the clinical balance and gait measures assessed at the beginning of the experiment.

The *slip outcome* was classified as backward loss of balance if the non-slipping recovery limb landed posterior to the forward slipping limb, and no loss of balance if the non-recovery limb landed anterior to the slipping limb [46]. The slip outcome was a dichotomous variable with “1” assigned for backward loss of balance and “0” for no loss of balance. The total number of participants who experienced balance loss in each group during each slip trial was assessed and represented as a percentage. *COM stability* (D-dimensionless) was calculated as the shortest distance from the instantaneous COM state (COM position and velocity) to the computation threshold against backwards loss of balance under slip conditions [35,47]. If the COM state was below the pre-established computational threshold (i.e., stability value < 0), it indicated a greater possibility of backward loss of balance. On the contrary, a positive COM stability value indicated a lower possibility of backward loss of balance. The COM kinematics (i.e., COM position and velocity) were computed from 3D-motion data using a 12-segment body representation [48]. The *COM position* was expressed relative to the rear edge of the base of support (BOS) (i.e., the slipping heel) by normalizing it to foot length. The *COM velocity* was also expressed relative to the rear edge of BOS (i.e., the slipping heel) and normalized by fraction of $\sqrt{g\times h}$ where ‘*g*’ represents acceleration of gravity and ‘*h*’ represents height of the participant. The COM stability was calculated at pre-slipping instance of slipping limb touchdown and post-slipping instance of recovery limb touchdown. These time events were detected by the force plate (based on each participant’s GRFs). During the perturbation trials, *maximum slip displacement* and *maximum slip velocity* were computed using the trajectory of the slider marker. Previous studies have shown that there is no relative motion between the participant’s heel and the movable plate (i.e., slider) marker during slipping [35,49]. Thus, the slider marker was used to calculate slipping limb kinematics in this study. *Maximum slip displacement* (meters) was calculated as the maximum distance travelled by the slider marker from the slip onset to the slipping foot lift off, and *maximum slip velocity* (meters/second) was calculated as the maximum value of the first order derivative of slip displacement in the same time period. *Compensatory step length* (meters) was calculated as the distance in anterior-posterior direction between the heel markers of the slipping and recovery limb at post-slipping instance of recovery limb touchdown [33].

#### 2.5.2. Secondary Outcome Measures

Secondary outcomes measures included clinical balance and gait assessments, i.e., functional mobility using Timed Up-and-Go test [50–52], dynamic balance using Berg Balance Scale [53,54], and gait speed using 10-Meter Walk Test [55].

### 2.6. Adaptation Characteristics

**Figure 1.**
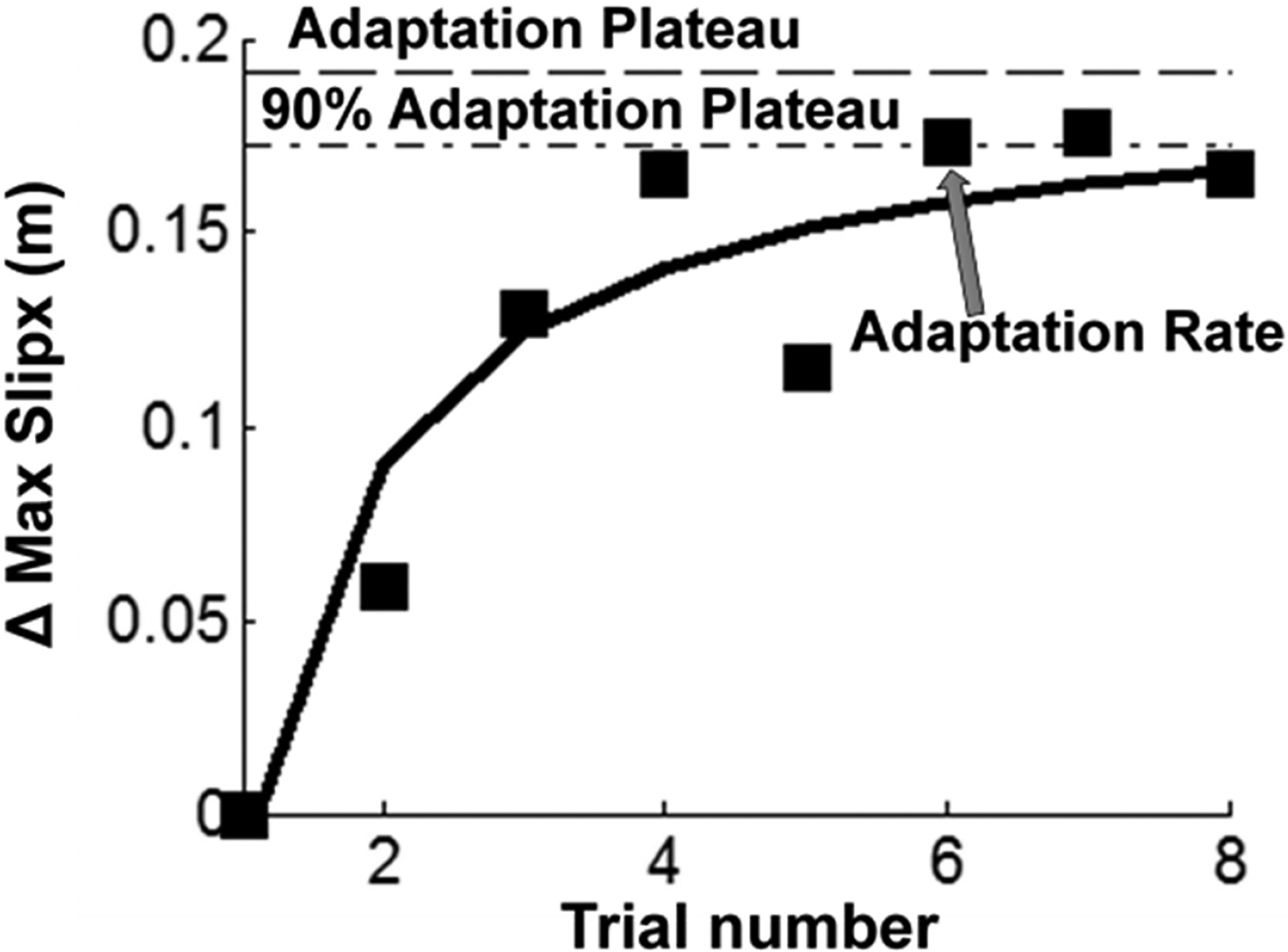
An example of nonlinear regression of adaptation curve for change in maximum slip displacement (Δ Max slipx) (m–meters) during repeated slip training (S1–S8) for a single participant. The adaptation plateau represents the best performance, which was derived from the nonlinear regression model, and the adaptation rate was defined as the number of trials required to reach 90% of the adaptation plateau. The square marker denotes the changes in slipping distance relative to S1, and the solid curve denotes the estimated adaptation curve. The dashed line denotes adaptation plateau achieved by the participant for maximum slip displacement during S1–S8. The dotted line denotes 90% of the adaptation plateau achieved by the participant for maximum slip displacement during S1–S8.

Two primary variables (*adaptation rate and adaptation plateau*) were used to compare the group differences in proactive and reactive balance adjustments to repeated slips [56–59]. Previous studies have reported that maximal improvements in motor performance occur at the beginning of the training block, followed by a performance plateau towards the end of the training block [27,35]. Thus, to characterize the adaptation curve for trial-to-trial performance (S1–S8), *adaptation plateau*, representing the amount of performance gains, was first calculated by using nonlinear regression to fit an inverse curve (y = a − b/x) [56]. Here ‘x’ denotes the trial number (1 to 8), and ‘y’ denotes the changes in these trials relative to S1, ‘a’ is the theoretical highest value, and ‘b’ is the adaptation slope. Thus, the adaptation plateau would theoretically represent a participant’s best performance achieved and the value of ‘a’ from the equation would best represent this variable. To quantify how fast could the adaptation be achieved, *adaptation rate* would subsequently be defined as the number of trials required to reach 90% of the adaptation plateau (>0.9a) (Figure 1). While previous studies that assessed these two variables named them *“learning rate”* and *“learning plateau”* [56–59], we will use the terms *“adaptation rate”* and *“adaptation plateau”* as motor learning typically occurs over a longer period of time (days, weeks, months or even years) [60–63] and our study only examines the short-term effect during a single session.

### 2.7. Statistical Analysis

The primary and secondary outcome measures were first assessed for their distribution using the Shapiro–Wilk test. The Shapiro–Wilk test indicated that normality assumption was met for all the primary and secondary outcome measures (*p* > 0.05). Demographics (age, height, and weight) and clinical outcome measures (Berg Balance Scale, Timed Up-and-Go Test, and 10-Meter Walk Test) were compared using a 1-way analysis of variance (ANOVA) across the 3 groups (young and older adults with chronic stroke and healthy older adults). A generalized estimating equations model (GEE) was used to determine the main effect of group (young and older adults with chronic stroke and healthy older adults), trial (S1–S8), and group × trial interaction on binary/dichotomous slip outcomes for slip adaptation, including backward or no loss of balance. A one-way analysis of variance (ANOVA) was performed for all kinematic variables in order to examine the main effect of group on adaptation plateau. Kruskal–Wallis test was performed to examine the group effect on adaptation rate. Significant effects (*p* < 0.05) were followed up with between-group pairwise comparisons using Tukey’s test of variances with Bonferroni corrections. All analyses were performed using SPSS version 25.0 (SPSS Inc., Chicago, IL, USA).

## 3. Results

### 3.1. Primary Outcome Measures

**Figure 2.**
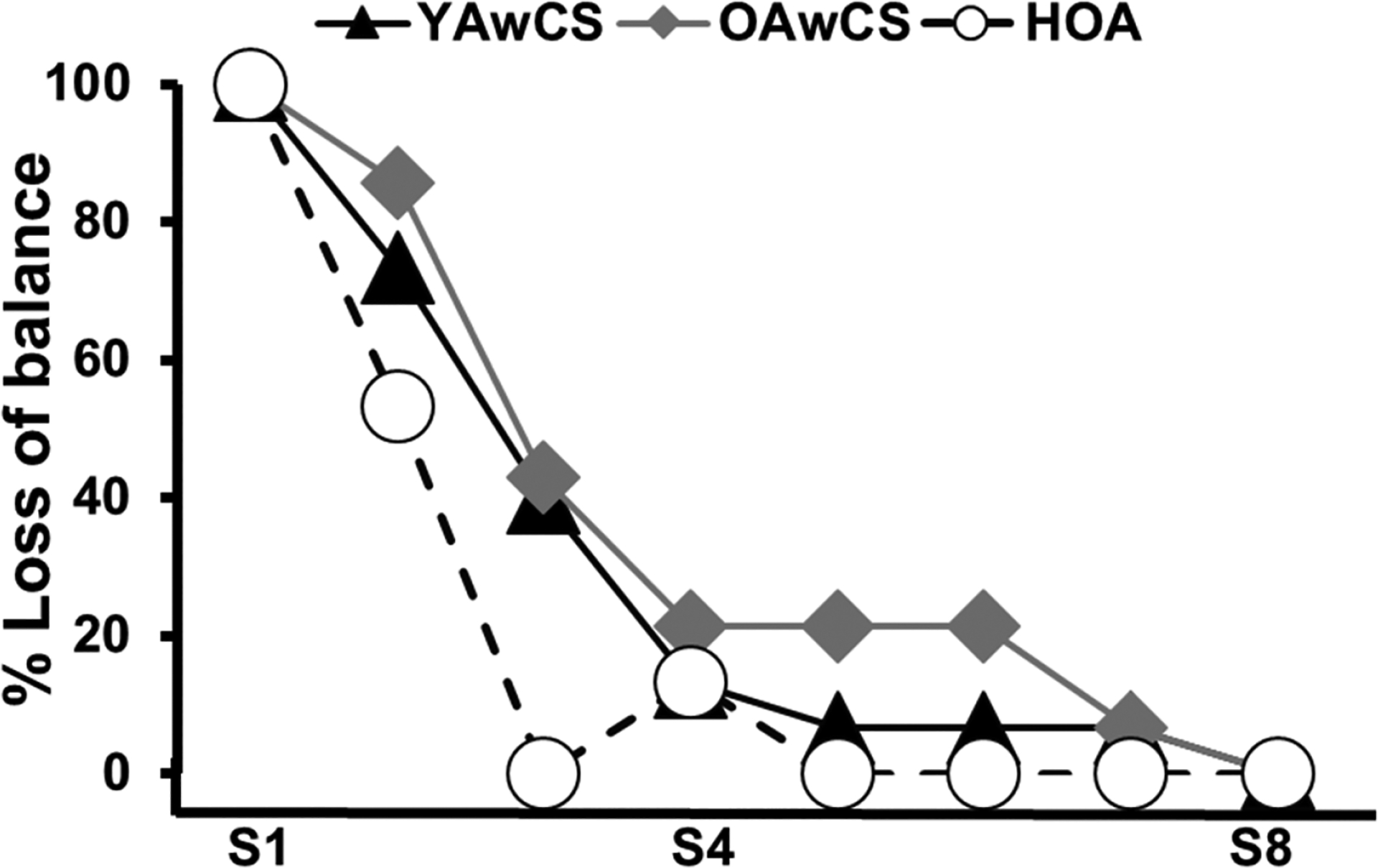
Trial-to-trial changes in percentage of backward balance losses for young adults with chronic stroke (YAwCS), older adults with chronic stroke (OAwCS) and healthy older adults (HOA) during a block of eight consecutive slips delivered to the non-paretic (for YAwCS and OAwCS)/dominant side (for HOA) (S1–S8). The slip outcome was a dichotomous variable with “1” assigned for backward loss of balance and “0” for no loss of balance. The total number of participants who experienced balance loss in each group during each slip trial is represented as a percentage in the figure. The figure shows that 100% of participants in each of the 3 groups (YAwCS, OAwCS and HOA) experienced loss of balance during the novel slip (S1). There were subsequent reductions in percentages of loss of balance with repeated perturbations such that 0% of participants in each of the 3 groups experienced loss of balance by the 8th slip (S8).

The GEE model demonstrated a main effect of group (*p* < 0.05) and trial (*p* < 0.05), and a group × trial interaction (*p* < 0.05) on balance loss (backward or no loss of balance) outcomes (Figure 2). All participants in all the 3 groups experienced a backward loss of balance (100%) during exposure to the novel slip (S1). Following S1, all 3 groups demonstrated reductions in balance losses such that all participants experienced no loss of balance (0%) on the last training trial (S8).

**Figure 3.**
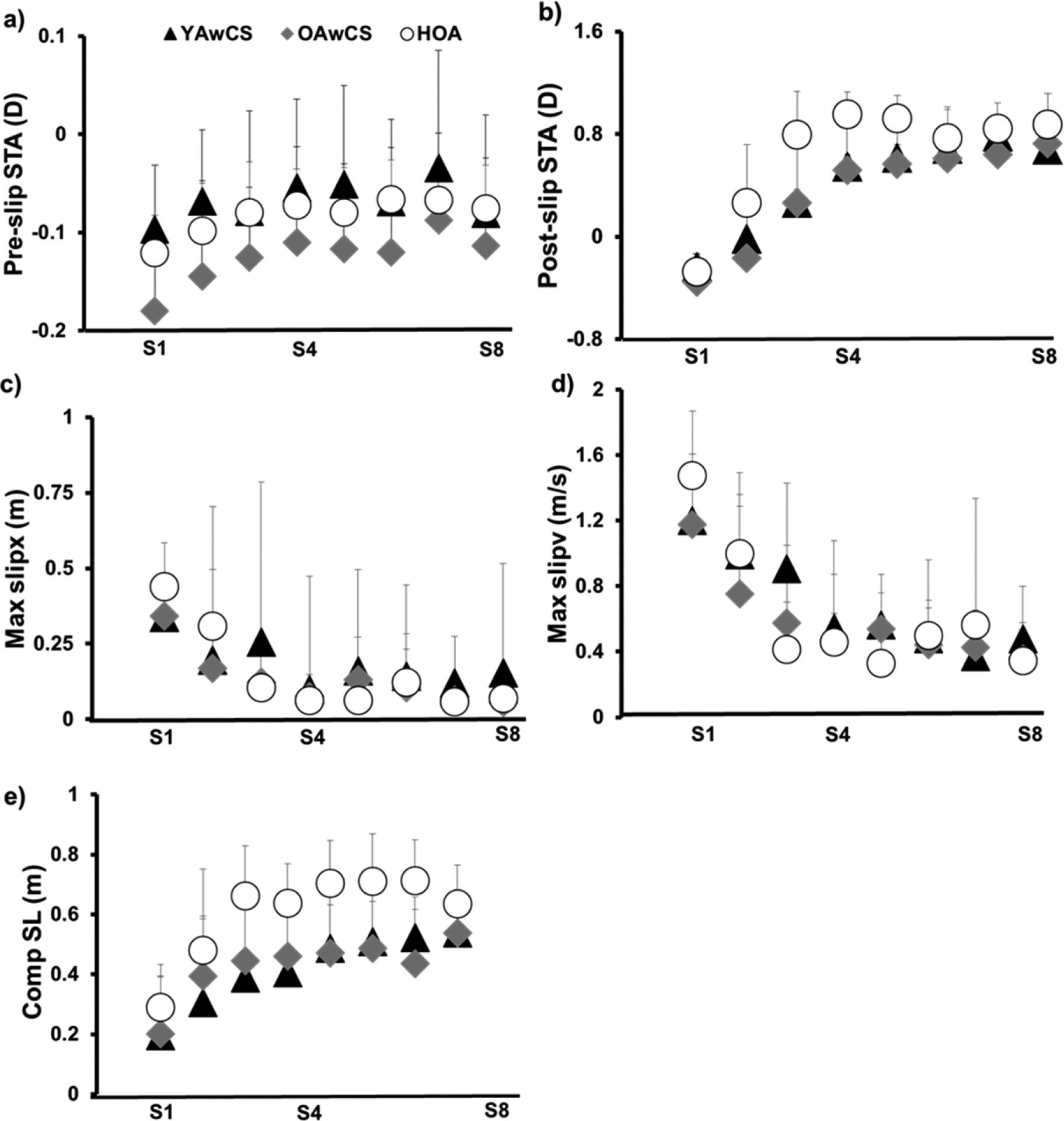
(**a**) Pre-slipping center of mass (COM) stability (Pre-Slip STA) (D–Dimensionless) computed at the instance of slipping limb touchdown; (**b**) Post-slipping center of mass (COM) stability (Post-Slip STA) (D–Dimensionless) computed at the instance of recovery limb touchdown; (**c**) Maximum slip displacement (Max slipx) (m–meters) measured from the instance of slip onset to the instance of slipping limb lift-off; (**d**) Maximum slip velocity (Max slipv) (m/s–meters/second) measured as the first derivate of maximum slip displacement from the instance of slip onset to the instance of slipping limb lift-off; (**e**) Compensatory step length (Comp SL) (m–meters) measured as the distance in anterior-posterior direction between the heel markers of the slipping and recovery limb at the instance of recovery limb touchdown; for young adults with chronic stroke (YAwCS), older adults with chronic stroke (OAwCS) and healthy older adults (HOA) during a block of eight consecutive slips delivered to the non–paretic (YAwCS and OAwCS)/dominant side (HOA) (S1–S8).

The key kinematic factors showed a non-linear adaptation curve (Figure 3); thus, the adaptation characteristics were extracted using non-linear regression models. The results showed a positive adaptation plateau (indicating improvements in variables) for all variables except for pre-slip COM velocity at the slipping limb touchdown, which only increased by 0.01 m/s (minimal change) in younger adults with chronic stroke and healthy older adults (Table 2). For the proactive factors (pre-slip COM position and COM stability), no group differences were found in the adaptation plateau and adaptation rate (*p* > 0.05) (Tables 2 and 3). For the reactive factors, no group effect was found in the adaptation rate (*p* > 0.05) (Table 3), while significant group effects were found in the adaptation plateau for post-slip COM stability, post-slip COM velocity, maximum slip displacement, and maximum slip velocity (*p* < 0.05 for all) (Table 2). Post-hoc comparisons revealed that both stroke groups (older and younger adults with chronic stroke) showed smaller adaptation plateaus for post-slip COM stability (*p* < 0.05), post-slip COM velocity (*p* < 0.05), and slip intensity (displacement and velocity, *p* < 0.05 for both) compared to healthy older adults. Further, both the stroke groups showed a trend of shorter compensatory steps than healthy older adults (*p* = 0.13). However, there were no significant differences in the adaptation plateaus of any variables between the two stroke groups (young and older adults with chronic stroke).

### 3.2. Secondary Outcome Measures

The one-way ANOVA showed significant group differences for age and all clinical measures (*p* < 0.05) (Table 1). Firstly, young and older adults with chronic stroke had significant differences in age and chronicity of stroke (*p* < 0.05). In addition, young adults and older adults with chronic stroke demonstrated lower scores on the Berg Balance Scale and Timed Up-and-Go Test compared to healthy older adults (*p* < 0.05). Further, older adults with chronic stroke displayed slower gait speed on the 10-Meter Walk Test compared to healthy older adults (*p* < 0.05). However, there were no significant differences in gait speed between young adults with chronic stroke and healthy older adults (*p* > 0.05).

## 4. Discussion

This study examined whether aging and cortical stroke can affect motor adaptation during exposure to a block of overground gait-slips delivered to the dominant/non-paretic limb. Contrary to our first hypothesis, the results showed that older adults with chronic stroke demonstrated a *similar adaptation rate* to their younger and healthy counterparts during repeated gait-slips. On the other hand, the results partially supported our second hypothesis and indicated a *smaller adaptation plateau* in both young and older adults with chronic stroke compared to healthy counterparts, while no differences in adaptation plateau were found between the two stroke groups.

All participants in each of the three groups demonstrated reductions in backward loss of balance (Figure 2) at a *similar adaptation rate* (Table 2) during exposures to gait-slips. Such reduction in balance losses were attributed to improvements in proactive and reactive balance variables (Figure 3a and Table 2). In-line with previous studies on healthy adults [32,35,64], all current study groups demonstrated improvements in pre-slip stability (Figure 3a) which were predominantly influenced by an anterior shift of COM position relative to the BOS (Table 2). Such changes in COM position with repeated slip exposures could arise from the recalibration of the internal representation of stability limits against the backward loss of balance, where the CNS updates its existing model or builds a new model in anticipation of upcoming slip(s) [65–67]. As previously shown in healthy adults [35,64,68], changes in pre-slip stability in all current study groups were accompanied by reactive balance adjustments (Figure 3b and Table 2). Specifically, young and older adults with chronic stroke in this study demonstrated reductions in slipping intensity (i.e., reduced slip displacement and reduced slip velocity) (Figure 3c,d and Table 2) with repeated slips at a similar rate as healthy older adults. Reductions in slipping intensity demonstrated by the current study groups could have primarily influenced improvements in post-slip COM stability, which is in parallel with previous studies [33,35]. Further, all groups demonstrated increases in compensatory step length with repeated slips at a similar rate (Figure 3e). Such changes in compensatory step length could have also resulted from reductions in slipping intensity thus leading to reductions in backward loss of balance which eliminated the need to execute a recovery step [27,33,35]. These results suggest that the presence of cortical stroke does not affect the ability of the CNS to acquire proactive and reactive adaptations to enhance COM stability during gait-slips for improving recovery outcomes and preventing balance loss.

It is well established that the cerebral cortex and cerebellum play an important role in balance and gait adaptations [69–74]. Studies have indicated that cerebellar stroke can impair the acquisition of motor skills during gait and balance-related tasks; however, this ability is shown to be intact in people with cortical stroke [70,75,76]. Previously, little was known about whether adults with cortical stroke could demonstrate adaptations during reactive balance tasks similarly to their healthy counterparts. Contrary to current study findings, previous studies showed that individuals with stroke demonstrate a reduced adaptation rate compared to healthy counterparts [76,77]. However, these studies included tasks such as split-belt gait adaptation and visuomotor adaptation which might not be perceived by the CNS as a high postural threat. With a reduced perceived threat, it is possible that such tasks allow the CNS greater time and flexibility for performance variability and error-based learning. Additionally, previous studies indicated that higher perturbation magnitude and greater postural threat, such as higher ground height, elicited higher cortical activations [78–81] and can accelerate changes in motor behavior [82–85]. Similarly, our current study included large-magnitude perturbations capable of inducing falls that mimicked real-life situations. Thus, both young and older adults with chronic stroke in this study might have perceived these perturbations with higher postural threat or might have experienced the penalties from losses of balance during the initial slips. Hence, to avoid potential injuries, their CNS was able to acquire, refine and update proactive and reactive balance variables that could reduce losses of balance at a similar rate as healthy older adults.

Despite the similar adaptation rate, both young and older stroke groups in this study demonstrated a *smaller adaptation plateau* for reactive balance variables compared to healthy older adults, including post-slip COM stability and slipping intensity (Table 3). Smaller improvements in post-slip stability could have primarily resulted from smaller performance gains particularly in post-slip COM velocity (Table 3). Previous studies showed that people with stroke exhibited reduced paretic muscle contraction power [86,87], reduced paretic lower limb muscular strength [35], and altered patterns of joint torques [88] and up to 20% of neuromuscular impairments exist even on the non-paretic side [89–91]. Similarly, reduced paretic knee extensor strength could have affected the propulsive impulse, indicated by changes in COM momentum, which was required to regain COM stability at recovery limb touchdown [35,92,93]. This could have resulted in a more posteriorly-directed COM velocity in both stroke groups compared to healthy older adults (Table 3). In addition, the smaller adaptation plateau for post-slip COM stability in young and older stroke groups could be influenced by smaller improvements in non-paretic slipping intensity (i.e., slip displacement and velocity) (Table 3). Previous literature on healthy adults have reported that slipping limb muscles (e.g., hip extensors) are important to attain slider control and reduce the intensity of a slip [94–97]. Thus, non-paretic limb deficits in both stroke groups might have affected the amount of reductions in slipping intensity (slip displacement and velocity) during non-paretic slips, as seen in this study (Figure 3c,d and Table 2). Thus, it is possible that bilateral stroke-related neuromuscular impairments might contribute to smaller adaptation plateaus in young and older adults with chronic stroke compared to healthy older adults in this study.

This study showed that regardless of age, people with chronic stroke acquired fall-resisting skills at a *similar adaptation rate* but a *smaller adaptation plateau* compared to healthy counterparts. The next logical question would be whether improving adaptation plateaus in people with chronic stroke would be functionally meaningful or have any additional practical benefits. Previous studies indicated that the process of motor adaptation involves two phases including a rapid, initial phase, which is postulated to be controlled by the cerebellum and typically sensitive to motor errors and vulnerable to memory decay [70,95,98]. This is followed by a slower, later phase, which is postulated to be controlled by the cerebral cortex and typically less sensitive to errors and resistant to memory decay [99,100]. Our results also demonstrated two phases of adaptation (Figure 3a–e), with no group differences in initial adaptation phase. However, our results indicated that the presence of cortical stroke can affect the later adaptation phase (*smaller adaptation plateau* in both stroke groups) (Table 3). It is possible that a smaller adaptation plateau might interfere with the future process of memory consolidation and could lead to greater memory decay over time, further hindering the process of motor skill retention or its transfer to real-life scenarios. Thus, improving the adaptation plateau might be an important component to induce neuroplastic changes that are resistant to memory decay. Given that reductions in adaptation plateaus could be attributed to bilateral impairments in people with stroke, training paradigms should consider impairment-oriented training that primarily focuses on restoring stroke-induced neuromuscular impairments [63,101–104]. Such intervention(s) could be used as supplemental agents along with PBT to enhance adaptation plateaus in individuals with chronic stroke. These supplemental agents might act as catalysts or primers for improving performance gains in reactive balance variables and might aid in the retention and/or carryover of acquired skills to real-life scenarios.

This study has some limitations. First, as the adaptation characteristics were estimated based on eight slip trials according to our study design, the small number of sample points might affect the accuracy of estimated regression coefficients for factors with larger error variance (i.e., for pre-slip COM stability). Second, although the current sample size was based on recommended guidelines for statistical considerations (at least 12 individuals/group) [105], this sample (n = 42) might not be large enough to infer the results to larger populations. Hence, the study results could be further validated with a larger clinical trial. Third, this study examined the age and stroke-related differences in motor adaptation characteristics to perturbations in a safe and controlled environment; hence, the translation of results to real-life settings must be done with caution. Fourth, the current study only focused on examining motor adaptation during non-paretic slips in young and older adults with chronic stroke. However, it is still unclear whether the paretic limb in people with chronic stroke can acquire the same fall-resisting skills as healthy counterparts using similar training paradigms. Further, the current study examined motor adaptation only during single-task conditions. It is possible that the addition of a concurrent cognitive task could affect the rate and characteristics of motor adaptation to overground gait-slips in young and older adults with chronic stroke. Fifth, all participants in both the stroke groups were all ambulatory individuals in their chronic phases of stroke recovery. Hence, the results cannot be generalized to sub-acute and acute phases of stroke recovery. Sixth, it is well established that executive functional impairment can impact motor task performance in community-dwelling older adults. Although the current study screened individuals for global cognitive function (via the Montreal Cognitive Assessment), future studies might consider including a more sensitive test for executive function assessment (e.g., the Trail Making Test [106]). Lastly, our primary aim was to assess age- and stroke-related biomechanical differences in motor adaptation; hence, the current study did not analyze associations between clinical and socio-demographic variables.

## 5. Conclusions

In this study, older adults with chronic stroke demonstrated a similar adaptation rate for proactive and reactive balance adjustments during gait-slips as their age-matched healthy and impairment-matched younger counterparts, indicating their intact ability to acquire short-term fall-resisting skills. However, contrary to healthy older adults, both young and older adults with chronic stroke showed a smaller adaptation plateau for reactive balance variables, suggesting that stroke-related impairments can reduce the amount of performance gains during repeated gait-slips. People living in chronic phases of stroke recovery, regardless of age, might benefit from supplemental agents that target bilateral neuromuscular impairments and can act as catalysts/primers for enhancing the amount of performance gains during repeated gait-slips.

## Figures and Tables

**Table 1.** Demographic characteristics and clinical outcomes of participants with their respective means and standard deviations.

Variables	Mean (SD)			*p* Value
	YAwCS	OAwCS	HOA	
Age,y	51.67 (7.05)	65.36 (4.16)	69.23 (5.17)	0.001^a,b^
Sex, M/F	10/5	12/2	7/6	0.61
Height, m	1.73 (0.08)	1.70 (0.11)	1.68 (0.15)	0.39
Weight, kg	80.40 (11.65)	82.52 (16.06)	76.10 (15.92)	0.51
Chronicity, y	7.2 (3.85)	13.4 (7.46)	N/A	0.01^a^
Type of stroke, H/I	5/10	8/6	N/A	0.36
AFO/No AFO	10/5	7/7	N/A	0.20
CMSA (Leg), (out of 7)	4.87 (1.06)	5.13 (0.74)	N/A	0.50
BBS (/56)	49.4 (3.60)	48.86 (6.19)	53.86 (1.96)	0.01^b,c^

Abbreviations: YAwCS: young adults with chronic stroke; OAwCS: older adults with chronic stroke; HOA: healthy older adults; y: years; m: meter; kg: kilogram; Type of stroke: H: hemorrhagic; I: ischemic; CMSA: Chedoke–McMaster Stroke Assessment scale; AFO: ankle foot orthosis; BBS: Berg Balance Scale; TUG: Timed Up-and-Go test; 10MWT: 10-Meter Walk Test; The *p*-values are results from the ANOVA for comparison between the three groups with significance level set at 0.05. For significant post-hoc pairwise comparisons—

a: significant group differences between YAwCS and OAwCS;

b: significant group differences between YAwCS and HOA;

c: significant group differences between OAwCS and HOA.

**Table 2.** Means and standard deviations of adaptation rate for all the kinematic variables. Kruskal–Wallis test was conducted to examine the group effect on the adaptation rate.

Adaptation Rate	Variables	Mean (SD)			Kruskal-Wallis	
		YAwCS	OAwCS	HOA	*p* Value	*χ*^2^ Value
Proactive	Pre-slip STA	3.07 (2.09)	3.64 (1.78)	3.54 (1.94)	0.56	1.16
	Pre-slip COMx	4.07 (2.02)	4.5 (2.03)	5.3 (1.65)	0.28	2.58
	Pre-slip COMv	2.2 (1.57)	2.29 (1.33)	2.46 (2.29)	0.74	0.59
	Post-slip STA	5.2 (1.82)	5.07 (1.69)	4. 54 (1.51)	0.52	1.3
	Post-slip COMx	5.47 (1.46)	4.43 (1.83)	4.77 (1.83)	0.15	3.75
Reactive	Post-slip COMv	4.2 (1.42)	4.5 (1.7)	4 (1.63)	0.54	1.23
	Max slipx	3.93 (2.01)	4 (1.62)	5.46 (1.94)	0.1	4.69
	Max slipv	4.67 (1.87)	4.79 (1.93)	4.62 (1.98)	0.95	0.1
	Comp SL	4.93 (1.94)	4.07 (2.43)	5.31 (2.36)	0.34	2.15

Abbreviations: SD: standard deviation; χ^2^: chi-square value; YAwCS: young adults with chronic stroke; OAwCS: older adults with chronic stroke; HOA: healthy older adults; COM: center of mass; COMx: center of mass position; COMv: center of mass velocity; STA: stability; Max slipx: maximum slip displacement; Max slipv: maximum slip velocity; Comp SL: Compensatory step length. The *p*-values are results from the Kruskal-Wallis test for comparison between the three groups.

**Table 3.** Means and standard deviations of adaptation plateau for all the kinematic variables. One-way ANOVA was conducted to examine the group effect on the adaptation plateau.

Adaptation Plateau	Variables	Mean (SD)			ANOVA *p* Value	F Value
		YAwCS	OAwCS	HOA		
Proactive	Pre-slip STA	0.04 (0.09)	0.08 (0.09)	0.06 (0.05)	0.57	0.57
	Pre-slip COMx	0.15 (0.11)	0.25 (0.23)	0.24 (0.17)	0.23	1.55
	Pre-slip COMv	0.01 (0.08)	0.05 (0.18)	0.01 (0.09)	0.62	0.49
	Post-slip STA	1.05 (0.33)	1.14 (0.26)	1.36 (0.16)	0.01^b,c^	4.95
	Post-slip COMx	1.89 (0.51)	1.7 (0.38)	1.89 (0.32)	0.039	0.95
Reactive	Post-slip COMv	0.24 (0.18)	0.25 (0.18)	0.43 (0.16)	0.01^b,c^	5.17
	Max slipx	0.23 (0.25)	0.31 (0.1)	0.43 (0.18)	0.03^b,c^	3.69
	Max slipv	0.83 (0.5)	0.94 (0.35)	1.28 (0.47)	0.03^b,c^	3.81
	Comp SL	0.36 (0.2)	0.34 (0.27)	0.49 (0.14)	0.13	2.11

Abbreviations: SD: standard deviation; ANOVA: analysis of variance; YAwCS: young adults with chronic stroke; OAwCS: older adults with chronic stroke; HOA: healthy older adults; COM: center of mass; COMx: center of mass position; COMv: center of mass velocity; STA: stability; Max slipx: maximum slip displacement; Max slipv: maximum slip velocity, Comp SL: Compensatory step length. The p-values are results from the ANOVA for comparison between the three groups with significance level set at 0.05. For significant post-hoc pairwise group comparisons—

a: significant group differences between YAwCS and OAwCS;

b: significant group differences between YAwCS and HOA;

c: significant group differences between OAwCS and HOA.
